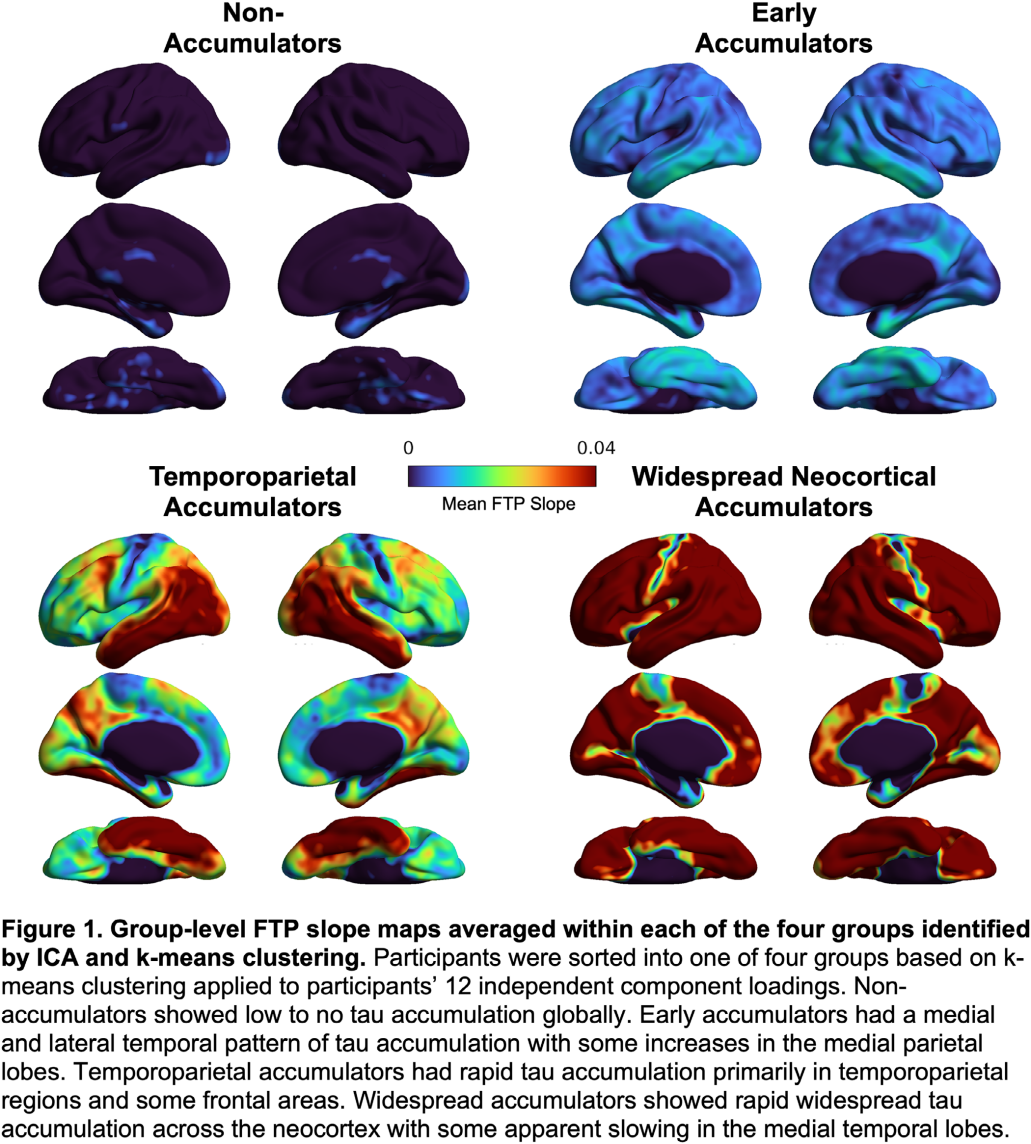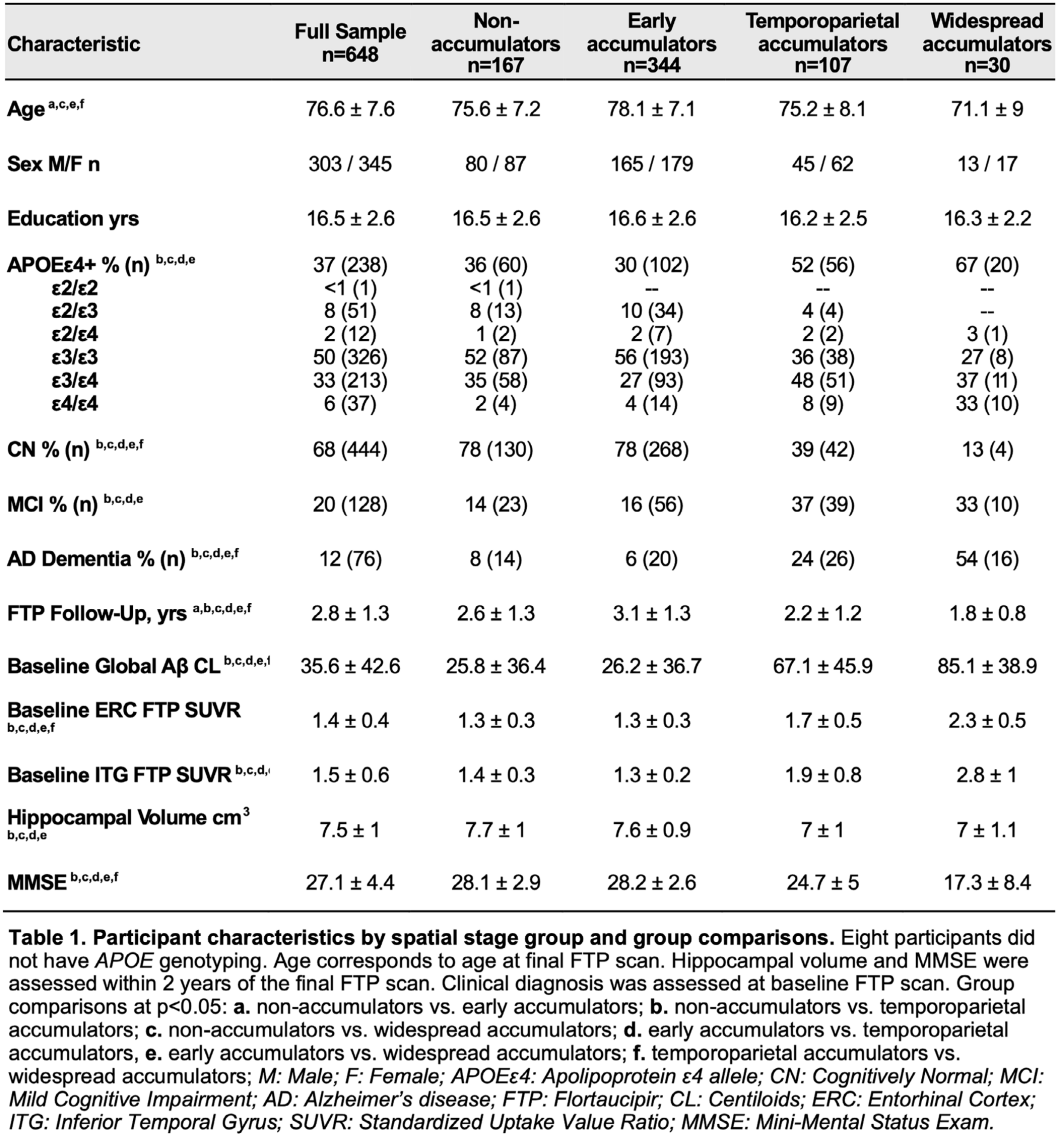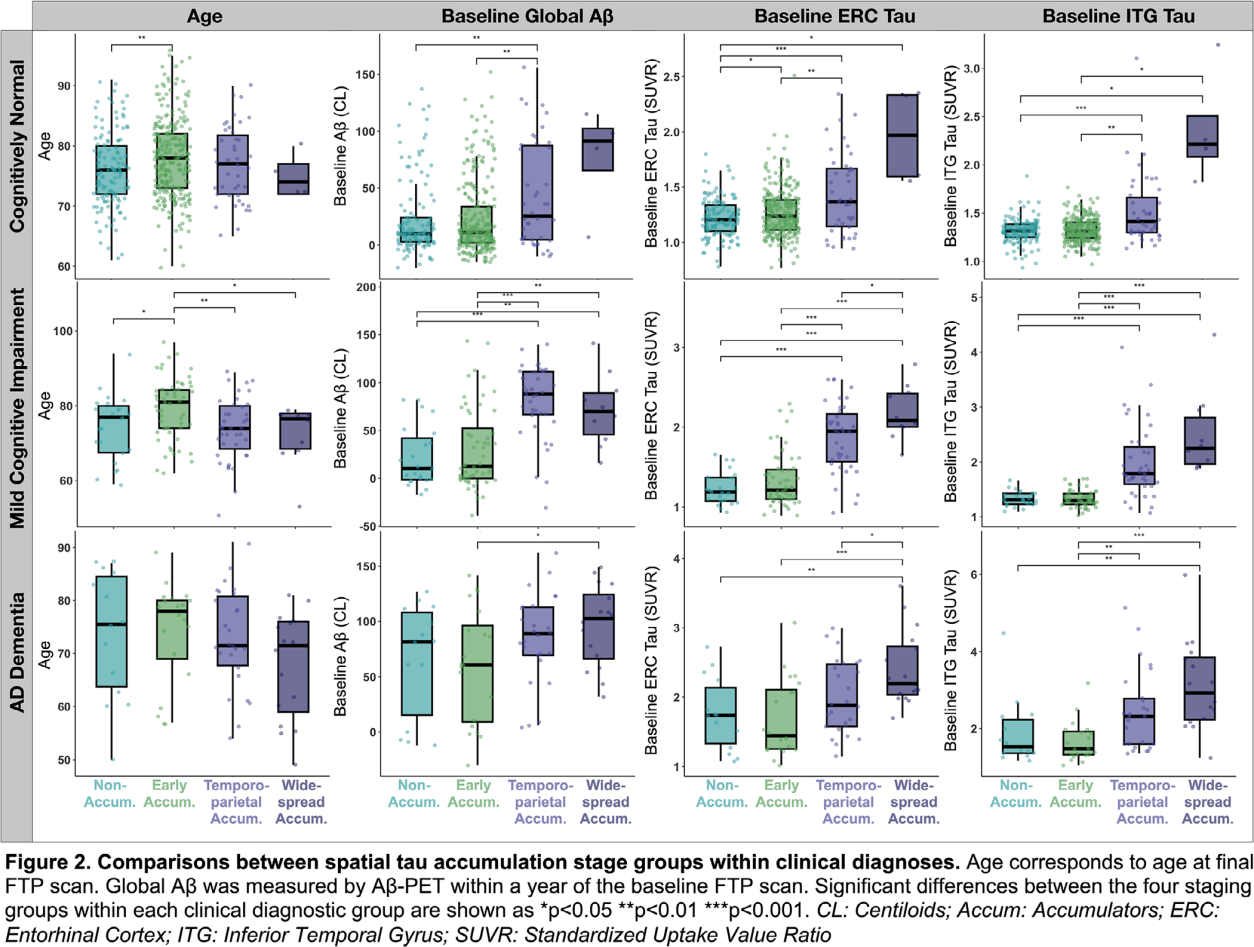# Data‐driven analysis of longitudinal tau‐PET scans reveals spatial patterns resembling tau accumulation stages

**DOI:** 10.1002/alz70862_109948

**Published:** 2025-12-23

**Authors:** Corrina S. Fonseca, Joseph Giorgio, Renaud La Joie, William J. Jagust, Theresa M. Harrison

**Affiliations:** ^1^ University of California, Berkeley, Berkeley, CA USA; ^2^ The University of Newcastle, Callaghan, NSW Australia; ^3^ Memory and Aging Center, Weill Institute for Neurosciences, University of California San Francisco, San Francisco, CA USA; ^4^ Department of Neurology, University of California, San Francisco, San Francisco, CA USA; ^5^ Lawrence Berkeley National Laboratory, Berkeley, CA USA; ^6^ Neuroscience Department, University of California, Berkeley, Berkeley, CA USA

## Abstract

**Background:**

Previous studies suggest there is variability in tau accumulation across the Alzheimer’s disease (AD) spectrum, but it remains unclear how these differences emerge and what drives heterogeneity. We used a voxel‐wise data‐driven approach to examine heterogeneity in spatial patterns of tau accumulation and how it relates to participant characteristics along the normal aging and clinical AD spectrum.

**Method:**

Whole‐brain voxel‐wise flortaucipir (FTP) SUVR slope maps were generated for 648 participants with 2 or more FTP scans from four independent studies: BACS, ADNI, HABS, and UCSF ADRC (1,568 FTP scans total). Spatial ICA was used to decompose the FTP slope maps into 30 independent components. After discarding 18 noise components, participants’ loadings were extracted for each of the remaining 12 components and k‐means clustering was applied to assign each participant to one of four clusters.

**Result:**

Group‐level FTP slope maps averaged within each cluster revealed four distinct tau accumulation staging groups: non‐accumulators, early accumulators, temporoparietal accumulators, and widespread neocortical accumulators (Figure 1). Participants with no or early tau accumulation were predominantly cognitively normal (CN) and Aβ‐, while the temporoparietal and widespread accumulator groups had a high proportion of Aβ+ MCI and AD dementia participants (Table 1). Widespread accumulators also had the highest prevalence of APOEe4 carriage and the lowest prevalence of APOEe2 carriage. Widespread accumulators were younger, had higher baseline Aβ, higher entorhinal and inferior temporal tau burden, and worse cognition than all other groups. Within the CN and MCI participants, early accumulators were older than non‐accumulating participants with the same clinical diagnosis (Figure 2). Within each clinical diagnostic group, temporoparietal and widespread accumulators had higher baseline AD pathology than their non‐ and early accumulating counterparts.

**Conclusion:**

Using a data‐driven approach, we identified four distinct patterns of tau accumulation resembling tau staging that were related to age, clinical diagnosis, baseline early AD pathology burden, and *APOE* genotype. Within the clinical diagnostic groups, participants with a more extensive accumulation pattern had higher baseline AD pathology. These findings are consistent with and add to previous literature that suggests heterogeneity in tau accumulation is associated with several between‐participant factors.